# Mobile App to Improve House Officers’ Adherence to Advanced Cardiac Life Support Guidelines: Quality Improvement Study

**DOI:** 10.2196/15762

**Published:** 2020-05-19

**Authors:** Vittal Hejjaji, Ali O Malik, Poghni A Peri-Okonny, Merrill Thomas, Yuanyuan Tang, David Wooldridge, John A Spertus, Paul S Chan

**Affiliations:** 1 Department of Cardiovascular Medicine Saint Luke's Mid America Heart Institute University of Missouri Kansas City Kansas City, MO United States; 2 Department of Internal Medicine University of Missouri Kansas City Kansas City, MO United States

**Keywords:** cardiac arrest, advanced cardiac life support, mHealth, quality improvement, medical education

## Abstract

**Background:**

Effective and timely delivery of cardiac arrest interventions during in-hospital cardiac arrest resuscitation is associated with greater survival. Whether a mobile app that provides timely reminders of critical interventions improves adherence to Advanced Cardiovascular Life Support (ACLS) guidelines among house officers, who may lack experience in leading resuscitations, remains unknown.

**Objective:**

The aim of this study was to assess the impact of a commercially available, dynamic mobile app on house officers’ adherence to ACLS guidelines.

**Methods:**

As part of a quality improvement initiative, internal medicine house officers were invited to participate and randomized to lead 2 consecutive cardiac arrest simulations, one with a novel mobile app and one without a novel mobile app. All simulations included 4 cycles of cardiopulmonary resuscitation with different cardiac arrest rhythms and were video recorded. The coprimary end points were chest compression fraction and number of correct interventions in each simulation. The secondary end point was incorrect interventions, defined as interventions not indicated by the 2015 ACLS guidelines. Paired *t* tests compared performance with and without the mobile app.

**Results:**

Among 53 house officers, 26 house officers were randomized to lead the first simulation with the mobile app, and 27 house officers were randomized to do so without the app. Use of the mobile app was associated with a higher number of correct ACLS interventions (out of 7; mean 6.2 vs 5.1; absolute difference 1.1 [95% CI 0.6 to 1.6]; *P*<.001) as well as fewer incorrect ACLS interventions (mean 0.3 vs 1.0; absolute difference –0.7 [95% CI –0.3 to –1.0]; *P*<.001). Simulations with the mobile app also had a marginally higher chest compression fraction (mean 90.9% vs 89.0%; absolute difference 1.9% [95% CI 0.6% to 3.4%]; *P*=.007).

**Conclusions:**

This proof-of-concept study suggests that this novel mobile app may improve adherence to ACLS protocols, but its effectiveness on survival in real-world resuscitations remains unknown.

## Introduction

### Background

Advanced Cardiovascular Life Support (ACLS) guidelines provide evidence-based algorithms to optimize the likelihood of survival in patients with in-hospital cardiac arrest (IHCA) [1]. Several ACLS components as well as the recent 2015 American Heart Association (AHA) resuscitation guidelines are critical for high-quality cardiopulmonary resuscitation (CPR) [2]. These include minimizing interruptions to chest compressions to achieve a high compression fraction, timely defibrillation, accurate and timely dosing of vasoactive drugs, and avoiding inappropriate ACLS treatments (eg, atropine for asystole). However, there is wide hospital-level variability in the delivery of timely defibrillation and epinephrine [3,4]. Whether a novel and portable mobile app that tracks critical interventions and provides real-time reminders and dosing guidance during a resuscitation improves adherence to ACLS guidelines remains unclear, but this would be important to understand as it may help reduce variability in the delivery of potentially lifesaving interventions.

Acute resuscitations are often chaotic, which may contribute to the variability in adherence to ACLS guidelines, thus attenuating the benefits of ACLS [5]. At many hospitals, this is compounded by the fact that house officers often lead resuscitations, although they may have little experience and feel inadequately prepared [6]. Use of an auxiliary mobile phone tool that includes real-time prompts for when to defibrillate or administer vasoactive medications, separate clocks for tracking chest compression duration and time since last defibrillation or vasoactive medication intervention, and dosing guidance for vasoactive medications (such as for epinephrine and amiodarone) may be an important adjunct to facilitate high-quality CPR among house officers. Moreover, such a tool has the potential to minimize interventions that are no longer recommended or even inappropriate per the current ACLS guidelines, such as procainamide or sodium bicarbonate.

### Objective

As part of a quality improvement program, we conducted a clinical trial wherein each house officer was randomized to perform a cardiac arrest simulation with and without a mobile app designed to support the use of ACLS guidelines. We examined whether use of the mobile app was associated with a higher number of correct ACLS interventions performed and chest compression fraction, as well as fewer inappropriate interventions, in a simulation setting.

## Methods

### Study Population and Setting

This study required all participants to perform 2 resuscitation simulations, one with and one without the ACLS mobile app. The study was a quality improvement initiative within the internal medicine residency program at the University of Missouri-Kansas City School of Medicine. All categorical and preliminary house officers were invited to participate by the residency director (DW). Participation was voluntary. A total of 53 ACLS-certified house officers out of 60 participated in the study between August 6, 2018, and September 7, 2018. The study was approved by the institutional review board at Saint Luke’s Hospital in Kansas City, Missouri (approved on November 4, 2018), as a quality improvement study. Regardless, individual informed consent was obtained for randomization and study participation.

**Figure 1:**
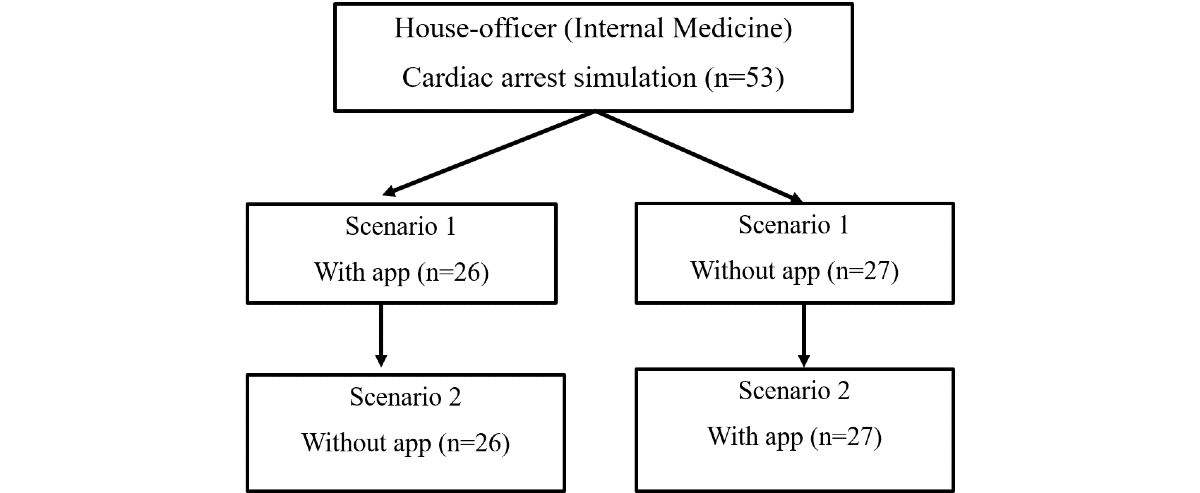
Scheme of randomization for leading each scenario with or without the mobile app.

Study simulations were conducted at an AHA-certified training center in Kansas City, Missouri, which frequently conducts mock codes to mimic a real-world inpatient setting. All house officer participants completed 2 different 10- to 12-min simulations consecutively—one with the mobile app and the other without the mobile app. Each of the 2 simulations comprised 4 cycles of CPR, rhythm identification, and defibrillation or drug administration, each cycle lasting for 2 min. A random number generator determined whether the house officer performed the first or second simulation with the mobile app tool (Figure 1).

**Figure 2:**
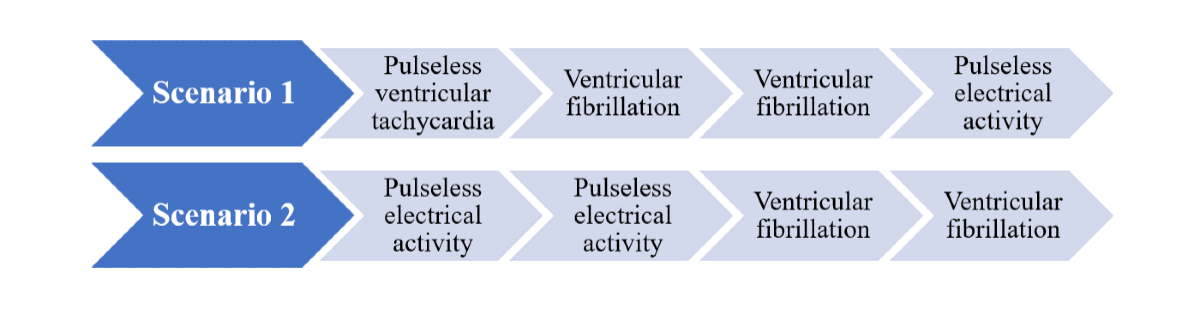
Scheme of cardiac arrest rhythms used in each scenario.

The different cardiac arrest rhythms used in each scenario are shown in Figure 2. Although the scenario in which the house officer would use the mobile app was decided randomly, the sequence of the 2 simulations itself varied depending on the day of the week on which the house officer participated in the study, such that half the days started with scenario 1 and the other half started with scenario 2.

All resuscitation equipment, such as the defibrillator, medications, intravenous lines, and airway, were obtained from a real-world crash cart. Two certified training personnel with predefined roles were assigned to each simulation. For data acquisition, both simulations (with and without the mobile app) were videotaped to ensure accurate documentation and timing of interventions for end point assessment.

### Study Intervention

The intervention of interest was a mobile app for ACLS delivery, which can be downloaded onto any smartphone and is compatible with both Android and iPhone operating system platforms. The Redivus Code Blue app was created and licensed by Redivus Health, and it is commercially available on Google Play and Apple App Store.

The goal of the mobile app is to offload team leader burden and simultaneously reduce delays in ACLS treatments, while increasing adherence to ACLS guidelines and AHA quality metrics. Activation of the *Code Blue* interface within the app initiates 2 sets of timers. One timer is for the CPR cycle and provides prompts for regular pulse checks at 2-min intervals, as recommended in ACLS. The second timer provides prompts for when the next defibrillation attempt or vasoactive medication dose should be administered, depending on the identified cardiac arrest rhythm (Multimedia Appendices 1 and 2 show the screenshots of the mobile app depicting the sequence of events). These time intervals are based on existing resuscitation guidelines (eg, 3 to 5 min between subsequent epinephrine doses). In addition to avoiding delays in administering treatments, the mobile app also provides guidance for the next recommended intervention and dosing in the ACLS algorithm to prevent deviation from guideline recommendations.

For this study, once informed consent was obtained, each participant was oriented to the mobile app through a 10-min prerecorded video, as well as to the simulation environment including personnel. All participants were permitted to use other decision aids (eg, ACLS algorithm card) they would normally use in either simulation to reflect usual care. They were then randomized to perform the first or second simulation with the mobile app.

### Study Outcomes

Our coprimary outcomes were overall compression fraction and the number of correct ACLS interventions. For the outcome of correct ACLS interventions, each of the 2 scenarios had a total of 7 correct interventions (defibrillation, epinephrine, and amiodarone). A correct intervention was defined as timely administration of the correct intervention (defibrillation or medication, including drug dose) per the ACLS protocol. If a correct intervention was skipped or the wrong medication dose was administered, a point was deducted from the total score of 7. In addition, we examined as a secondary outcome, the number of incorrect interventions, defined as an inappropriate ACLS treatment based on cardiac arrest rhythm. Examples of incorrect treatment include defibrillation for a nonshockable cardiac arrest rhythm and medications not recommended in the current ACLS guidelines (eg, atropine, bicarbonate, and procainamide).

### Statistical Analysis

Each simulation recording was viewed and graded by research personnel, equally divided among the 4 individuals, with 10% of the simulations validated by a second reviewer, who separately graded the simulation to ensure accuracy and consistency in scoring. Discordant ratings between 2 graders were resolved by a third researcher (PC). Each research personnel grading the simulations was provided with a structured score sheet and instructions detailing the use of the score sheet. Compression fraction was defined as the total amount of time (in seconds) taken for performing CPR divided by the total time of the simulation, and this was obtained from the time stamp for each period in the simulation scenario when CPR was and was not being administered. Similarly, for the number of correct ACLS interventions, we used the time stamp to ensure timely delivery of medications and defibrillations. For epinephrine and amiodarone dosing, where the recommended interval is 3 to 5 min between doses, we allowed a 15-second grace window on either side of this time interval and graded all medication doses ordered between 2:45 and 5:15 min as correct, as long as the medication dosing was also correct (1 mg of epinephrine; 300 mg and 150 mg for the first and second doses of amiodarone, respectively) and as long as it was the appropriate treatment for the identified rhythm.

For the coprimary outcomes, we used a paired *t* test to assess differences. For the secondary outcome of incorrect number of interventions, we used the Wilcoxon signed-rank test to estimate the effect of the mobile app on ACLS adherence. In addition, we conducted additional interaction analyses to assess whether the study outcome results differed by the house officer’s level of training (first vs second vs third year), whether the mobile app was used for the first or the second simulation to rule out a “learning effect,” and whether the house officer had previous experience with leading a real-world resuscitation event in the hospital. To accomplish this, we constructed 3 multivariable models with correct and incorrect interventions as the outcome and separately evaluated interaction terms between use of the mobile app and these 3 study factors, using linear regression. All analyses were performed using SAS version 9.4 (SAS institute, Cary, North Carolina, United States) and were evaluated at a two-sided significance level of .05.

## Results

### User Statistics

Of the 53 participants in the study, 24 (45%) participants were first-year, 15 (28%) participants were second-year, and 14 (26%) participants were third-year house officers. Approximately half of the (26/53, 49%) house officers were randomized to use the mobile app for their first simulation. A total of 10 house officers (10/53, 19%) had previously led at least one real-world resuscitation for cardiac arrest in the hospital.

### Evaluation Outcomes

Overall, use of the mobile app resulted in a small improvement in compression fraction (mean 90.9% vs 89.0%; absolute difference 1.9% [95% CI 0.6% to 3.4%]; *P*=.007; Table 1).

**Table 1:** Effect of the mobile app on study outcomes.

Outcome variable	Without the app, mean (SD)	With the app, mean (SD)	Absolute difference with the app, (95% CI)	*P* value
Compression fraction	89.0% (5.0%)	90.9% (2.3%)	1.9% (0.6% to 3.4%)	.007
Number of correct interventions	5.1 (1.6)	6.2 (1.1)	1.1 (0.6 to 1.6)	<.001
Number of incorrect interventions	1.0 (1.3)	0.3 (0.6)	−0.7 (−0.3 to −1.0)	<.001

The number of correct ACLS interventions (out of 7) was significantly higher among simulations in which the mobile app was used (mean 6.2 vs 5.1; absolute difference 1.1 [95% CI 0.6 to 1.6]; *P*<.001). The reasons for not receiving credit for a correct intervention during the simulations, stratified by mobile app use, are outlined in Table 2.

**Table 2:** Most common reasons for not performing a correct Advanced Cardiovascular Life Support intervention.

Reason for missing intervention			Total, N	Without the app, n	With the app, n
**Epinephrine**					
	**Incorrect epinephrine timing**				
		First dose	15	13	2
		Second dose	27	20	7
**Amiodarone**					
	Failed to give second dose of amiodarone		28	19	9
	Failed to give any dose of amiodarone		17	13	4
	**Incorrect amiodarone dose**				
		First dose	8	6	2
		Second dose	5	1	4
	Incorrect amiodarone timing		10	5	5
**Chest compressions**					
	Pulse check at irregular intervals affecting overall compression fraction		7	7	0

The most common reasons for missing a correct ACLS intervention were incorrect timing of epinephrine dose (n=42) and not administering amiodarone at all when indicated by ACLS guidelines (n=45). These occurred more commonly in simulations performed with usual care than with the mobile app.

For our secondary outcome, use of the mobile app also resulted in a fewer number of incorrect interventions (mean 0.3 vs 1.0; absolute difference −0.7 [95% CI −0.3 to −1.0]; *P*<.001). The most common reasons for an incorrect intervention are summarized in Table 3.

**Table 3:** Most common incorrect Advanced Cardiovascular Life Support interventions performed.

Incorrect intervention	Total, N	Without the app, n	With the app, n
Incorrect rhythm identification	17	10	7
Incorrect administration of epinephrine and failure to defibrillate pulseless ventricular tachycardia/ventricular fibrillation	13	10	3
Inappropriate defibrillation for PEA^a^	8	7	1
Used atropine to treat PEA	7	5	2
Checked blood pressure during chest compressions	6	3	3

^a^PEA: pulseless electrical activity.

The most common reasons included incorrect rhythm identification (n=17), incorrectly administering epinephrine and not performing defibrillation for a shockable cardiac arrest rhythm (n=13), inappropriate defibrillation for a nonshockable pulseless electrical activity (PEA) rhythm (n=8), and use of atropine for a PEA cardiac arrest rhythm (n=7). Use of the mobile app resulted in a numerically lower number of each of these incorrect interventions.

Finally, there were no significant interactions (all *P* values for interaction were >.31) between the use of the mobile app and the year of house officer training, whether the mobile app was used for the first or second simulation, and whether the participant had previously led a resuscitation in the hospital setting for either the end point of the number of correct interventions or the number of incorrect interventions (Table 4).

After the completion of the 2 simulations, participants were given the opportunity to describe their experience using the mobile app, and the most common comments reported by 2 or more participants are summarized in Textbox 1.

**Table 4:** Interaction analyses for the end points of total correct interventions and total incorrect interventions.

Interaction variables			*P* value
**Number of correct interventions**			
	App usage x house officer training level	>.99	
	App usage x sequence^a^	.32	
	App usage x previous experience in leading codes	.50	
**Number of incorrect interventions**			
	App usage x house officer training level	.50	
	App usage x sequence^a^	.31	
	App usage x previous experience in leading codes	.52	

^a^Indicates the sequence of simulations (whether mobile app was used with the first or second simulation).

**Qualitative data from the participants.**

House officers’ comments postintervention:

1. Advantages
  “The app was most useful for timing of cycles and Epi intervals”
  “Good experience. I like the functionality of the app. If the documents section can be integrated into the existing EMR so that the physician can look at comorbidities and history that would be very helpful.”
  “I could definitely see an improvement in adhering to the guidelines using the app. At our institution nursing staff keeps track of time. Having to keep time threw me off a little during the simulation without the app. Overall it was a great experience using the app”
  “If you don’t remember the sequence the app prompts you which is great. If you do remember the sequence the app still keeps all the timing appropriate and code running smoothly”
  “Although this study was among resident doctors due to the fact that it is currently expected of all doctors to be well versed in running code blues, with apps like these it should be possible for any health care professional to successfully run a code blue”
2. Limitations/suggestions for improvement
  “I would strongly suggest use vibratory cues for the timing. I found myself looking down too much at the app and not enough at assessing clinical status”
  “My only concern with the app is for some medications, it doesn’t specify the dose until it is time for it to be given. It would be nice if the amount of the next dose was displayed, so you could ready it in advance”

## Discussion

### Principal Findings

In this quality improvement trial to improve the rates of ACLS adherence among internal medicine house officers in simulations of IHCAs, we evaluated the impact of a dynamic mobile app that provides timely reminders and dosing guidance for ACLS interventions to the resuscitation team leader. Using each participant as their own control, we found that the use of this mobile app increased overall compression fraction and the number of correct interventions. Moreover, the use of the mobile app resulted in 19 fewer occasions of incorrect interventions delivered. In addition, the results for all 3 outcomes were not different, regardless of the house officer’s year of training, previous experience with leading in-hospital resuscitations, and whether the app was used for the first or second simulation. This study’s findings suggest that the use of novel assistive technologies such as this mobile app could improve adherence to ACLS guidelines in hospitalized patients with cardiac arrest.

### Comparison With Previous Work

To the best of our knowledge, this is the first study in the United States to have evaluated the use of a cardiac arrest mobile app among house officers. Most teaching hospitals have resuscitation teams structured around house officers being the team leader [7]. The chaotic and often disorganized environment of an emergent cardiac arrest resuscitation, as well as house officers’ infrequent exposure to leading resuscitations, makes house officers particularly suitable candidates for the use of mobile apps, such as the one used in this study, to minimize team leader burden. Dynamic decision aids that can reduce this burden on physician trainees have the potential to improve resuscitation care by increasing ACLS adherence and the overall quality of CPR delivered [8]. Improving adherence and standardizing care are important, as delays in the administration of defibrillation for a shockable rhythm and epinephrine for a nonshockable rhythm have been associated with worse survival outcomes for IHCA [9-11]. Furthermore, ACLS and the 2015 AHA resuscitation guidelines have highlighted the importance of effective chest compressions throughout the resuscitation process; therefore, minimizing interruptions to chest compressions should be a primary goal of all resuscitations [12].

Overall, this study found that house officers achieved a 22% relative increase in the number of correct ACLS interventions (6.2 vs 5.1). We found that the use of the mobile app ensured proper timing and dosing of the vasoactive medications, epinephrine and amiodarone, and failure to do so was the most common reason for missing a correct ACLS intervention in this study (see Table 2). Less robust results (15% relative increase) were observed in another study examining the use of a different electronic decision support tool among fourth-year medical students [8]. Moreover, this other study was conducted before the 2015 AHA/ACLS update, which delineated the importance of compression fraction and recommended an average fraction of more than 60% to be ideal [13]. Although this study showed a small improvement in compression fraction with the use of the mobile app, it remained above 60% and, more importantly, was not lower than that with usual care, as there may be concerns that decision tools may increase the frequency of interruptions to chest compressions [14].

Beyond improving adherence to correct ACLS interventions, we also found that it decreased the delivery of incorrect interventions. Inaccurate rhythm identification by house officers was the most common error and an issue for which the mobile app provides no benefit, as the app relies on the house officer to correctly identify the cardiac arrest rhythm to trigger the proper algorithm. We found that there were 10 inaccurate rhythm identifications in simulations without the mobile app and 7 inaccurate rhythm identifications with the app. The other reasons for an incorrect ACLS intervention were areas for which the mobile app can provide benefit. Administering epinephrine without defibrillating a shockable cardiac arrest rhythm or defibrillating a nonshockable rhythm clearly indicates difficulty of the team leader to recall the correct ACLS algorithm, and this was more common in simulations without the mobile app than with its use (see Table 3). Given that the use of the mobile app significantly reduced the number of potentially life-threatening incorrect interventions, we believe it to be extremely useful in a clinical setting, but further studies with real-world use of the app will determine if the differences noticed in this analysis are clinically relevant.

### Limitations

This study should be interpreted in the context of several limitations. First, the mobile ACLS app was evaluated in a controlled simulation setting, and its use and impact during emergent hospital resuscitations were not evaluated. As such, this trial is a proof-of-concept study of the mobile app’s value, but it will require further validation in real-world settings. This is particularly important because the potential benefits of using a mobile app are dependent on the app being used and the frequency and consistency of launching the app, and any delays in doing so are unknown and need to be examined in future studies. Moreover, the impact of the mobile app on overall survival rates for IHCA is unknown. Second, other components of resuscitation quality, such as ventilation and chest compression depth and rate, are not influenced by the mobile app. Therefore, the mobile app is only able to affect some, but not all, aspects of emergent resuscitation care. Third, this mobile app was evaluated among house officers only, and its benefits may not be generalizable to more experienced physicians. It is possible that experienced physicians have fewer gaps in the quality of their acute resuscitation care and that the mobile app would have little benefit. It is also possible that house officers attend more acute resuscitations in the hospital than other physicians and that the mobile app may show even greater benefit in physicians who have long completed their training. Fourth, this was a single-center study, and the findings may not reflect practice patterns at other institutions. Finally, the mobile app was designed to reduce team leader burden, but it has no direct effect on the team leader’s leadership skills, which can play an important role in resuscitation care variability.

### Conclusions

The use of a novel mobile app by house officers during simulations for IHCA was associated with better adherence to ACLS performance measures, a lower rate of incorrect interventions, and a mildly higher chest compression fraction. Given these promising findings, further testing in real-world care should be conducted.

## Appendix

Multimedia Appendix 1 Mobile app screenshots depicting the sequence of events for a nonshockable rhythm. A: The appropriate rhythm type is selected by the user. B: The app prompts the user to use epinephrine. C: Once epinephrine is delivered, a separate timer starts for the next dose of epinephrine. VFIB: ventricular fibrillation; VTACH: ventricular tachycardia; PEA: pulseless electrical activity; AED: automated external defibrillator; Defib: defibrillation.

Multimedia Appendix 2 Mobile app screenshots depicting the sequence of events for a shockable rhythm. A: The appropriate rhythm type is selected by the user. B: The app prompts the user to use the defibrillator by following steps 1 to 4, thereby avoiding chest compression interruptions during defibrillation. C: Once shock is delivered, a separate timer starts for the next dose of amiodarone. VFIB: ventricular fibrillation; VTACH: ventricular tachycardia; PEA: pulseless electrical activity.

Multimedia Appendix 3 CONSORT-eHEALTH checklist (V 1.6.1).

## Abbreviations

ACLS: Advanced Cardiovascular Life Support
AHA: American Heart Association
CPR: cardiopulmonary resuscitation
IHCA: in-hospital cardiac arrest
PEA: pulseless electrical activity

## References

[1] McEvoy MD, Field LC, Moore HE, Smalley JC, Nietert PJ, Scarbrough SH (2014). The effect of adherence to ACLS protocols on survival of event in the setting of in-hospital cardiac arrest. Resuscitation.

[2] Link MS, Berkow LC, Kudenchuk PJ, Halperin HR, Hess EP, Moitra VK, Neumar RW, O'Neil BJ, Paxton JH, Silvers SM, White RD, Yannopoulos D, Donnino MW (2015). Part 7: Adult Advanced Cardiovascular Life Support: 2015 American Heart Association Guidelines Update for Cardiopulmonary Resuscitation and Emergency Cardiovascular Care. Circulation.

[3] Khera R, Chan PS, Donnino M, Girotra S, American Heart Association’s Get With The Guidelines-Resuscitation Investigators (2016). Hospital variation in time to epinephrine for nonshockable in-hospital cardiac arrest. Circulation.

[4] Chan PS, Nichol G, Krumholz HM, Spertus JA, Nallamothu BK, American Heart Association National Registry of Cardiopulmonary Resuscitation (NRCPR) Investigators (2009). Hospital variation in time to defibrillation after in-hospital cardiac arrest. Arch Intern Med.

[5] Merchant RM, Berg RA, Yang L, Becker LB, Groeneveld PW, Chan PS, American Heart Association's Get With the Guidelines-Resuscitation Investigators (2014). Hospital variation in survival after in-hospital cardiac arrest. J Am Heart Assoc.

[6] Hayes CW, Rhee A, Detsky ME, Leblanc VR, Wax RS (2007). Residents feel unprepared and unsupervised as leaders of cardiac arrest teams in teaching hospitals: a survey of internal medicine residents. Crit Care Med.

[7] Lauridsen KG, Schmidt AS, Adelborg K, Løfgren B (2015). Organisation of in-hospital cardiac arrest teams - a nationwide study. Resuscitation.

[8] Field LC, McEvoy MD, Smalley JC, Clark CA, McEvoy MB, Rieke H, Nietert PJ, Furse CM (2014). Use of an electronic decision support tool improves management of simulated in-hospital cardiac arrest. Resuscitation.

[9] Chan PS, Krumholz HM, Nichol G, Nallamothu BK, American Heart Association National Registry of Cardiopulmonary Resuscitation Investigators (2008). Delayed time to defibrillation after in-hospital cardiac arrest. N Engl J Med.

[10] Donnino MW, Salciccioli JD, Howell MD, Cocchi MN, Giberson B, Berg K, Gautam S, Callaway C, American Heart Association’s Get With The Guidelines-Resuscitation Investigators (2014). Time to administration of epinephrine and outcome after in-hospital cardiac arrest with non-shockable rhythms: retrospective analysis of large in-hospital data registry. Br Med J.

[11] Patel KK, Spertus JA, Khariton Y, Tang Y, Curtis LH, Chan PS, American Heart Association’s Get With the Guidelines–Resuscitation Investigators (2018). Association between prompt defibrillation and epinephrine treatment with long-term survival after in-hospital cardiac arrest. Circulation.

[12] Meaney PA, Bobrow BJ, Mancini ME, Christenson J, de Caen AR, Bhanji F, Abella BS, Kleinman ME, Edelson DP, Berg RA, Aufderheide TP, Menon V, Leary M, CPR Quality Summit Investigators‚ the American Heart Association Emergency Cardiovascular Care Committee‚ the Council on Cardiopulmonary‚ Critical Care‚ Perioperative and Resuscitation (2013). Cardiopulmonary resuscitation quality: [corrected] improving cardiac resuscitation outcomes both inside and outside the hospital: a consensus statement from the American Heart Association. Circulation.

[13] Neumar RW, Shuster M, Callaway CW, Gent LM, Atkins DL, Bhanji F, Brooks SC, de Caen AR, Donnino MW, Ferrer JM, Kleinman ME, Kronick SL, Lavonas EJ, Link MS, Mancini ME, Morrison LJ, O'Connor RE, Samson RA, Schexnayder SM, Singletary EM, Sinz EH, Travers AH, Wyckoff MH, Hazinski MF (2015). Part 1: Executive Summary: 2015 American Heart Association Guidelines Update for Cardiopulmonary Resuscitation and Emergency Cardiovascular Care. Circulation.

[14] Nelson KL, Shilkofski NA, Haggerty JA, Saliski M, Hunt EA (2008). The use of cognitive AIDS during simulated pediatric cardiopulmonary arrests. Simul Healthc.

