# Supplementary figures and images for: Mobile App to Improve House Officers’ Adherence to Advanced Cardiac Life Support Guidelines: Quality Improvement Study
Source: JMIR Mhealth Uhealth. 2020 May 19;8(5):e15762. doi: 10.2196/15762 (PMC7267993; doi:10.2196/15762)

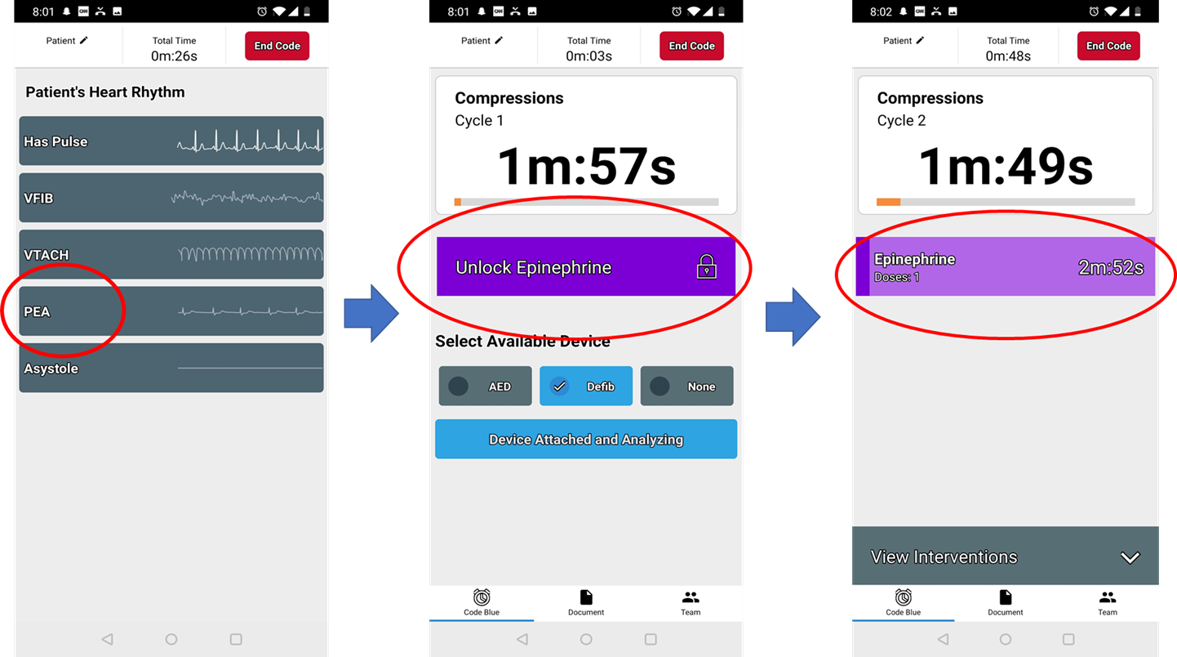

Supplement: Multimedia Appendix 1 [file mhealth_v8i5e15762_app1.png]

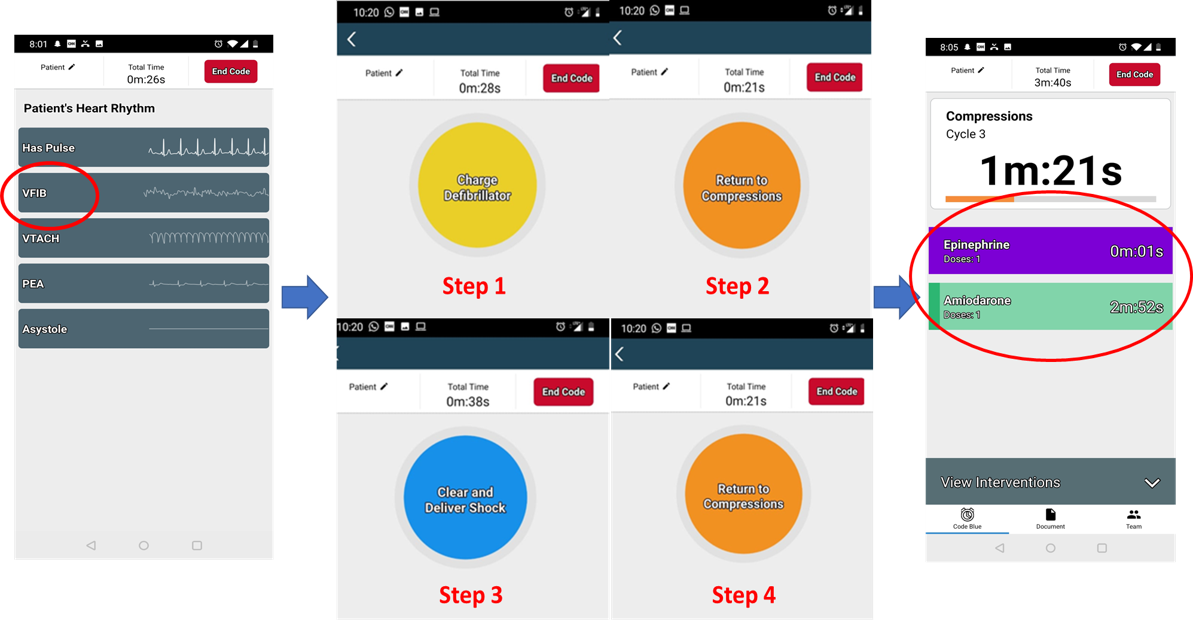

Supplement: Multimedia Appendix 2 [file mhealth_v8i5e15762_app2.png]
